# Symbiotic bacteria, immune-like sentinel cells, and the response to pathogens in a social amoeba

**DOI:** 10.1098/rsos.230727

**Published:** 2023-08-16

**Authors:** Trey J. Scott, Tyler J. Larsen, Debra A. Brock, So Yeon Stacey Uhm, David C. Queller, Joan E. Strassmann

**Affiliations:** Department of Biology, Washington University in St. Louis, St. Louis, MO 63130, USA

**Keywords:** sentinel cells, symbiosis, pathogens, *Dictyostelium*, *Paraburkholderia*

## Abstract

Some endosymbionts living within a host must modulate their hosts' immune systems in order to infect and persist. We studied the effect of a bacterial endosymbiont on a facultatively multicellular social amoeba host. Aggregates of the amoeba *Dictyostelium discoideum* contain a subpopulation of sentinel cells that function akin to the immune systems of more conventional multicellular organisms. Sentinel cells sequester and discard toxins from *D. discoideum* aggregates and may play a central role in defence against pathogens. We measured the number and functionality of sentinel cells in aggregates of *D. discoideum* infected by bacterial endosymbionts in the genus *Paraburkholderia*. Infected *D. discoideum* produced fewer and less functional sentinel cells, suggesting that *Paraburkholderia* may interfere with its host's immune system. Despite impaired sentinel cells, however, infected *D. discoideum* were less sensitive to ethidium bromide toxicity, suggesting that *Paraburkholderia* may also have a protective effect on its host. By contrast, *D. discoideum* infected by *Paraburkholderia* did not show differences in their sensitivity to two non-symbiotic pathogens. Our results expand previous work on yet another aspect of the complicated relationship between *D. discoideum* and *Paraburkholderia*, which has considerable potential as a model for the study of symbiosis.

## 1. Introduction

Microbes live in a world replete with other microbes with which they must interact. The most intimate interactions are symbioses, in which unlike organisms live closely associated with or even inside of one another. Symbioses can have many different effects on the participants' fitness, abilities and evolutionary fate. Many symbioses enable organisms to survive in ways otherwise beyond them—some symbionts expand the resources their partners can use [1,2], increase their resistance to abiotic stress [3], or protect them from hostile organisms [4,5]. Some of the most dramatic examples of symbiosis have had enormous impacts on the history of life, from enabling the development of complex multicellular organisms to shifting the composition of the planet's atmosphere on a grand scale [6–8].

However, the line between friend and foe can be blurry. Wherever organisms come together, there will be conflict, even within the most intimate and long-lasting friendships [9,10]. Many beneficial symbioses are thought to have evolved from initially antagonistic relationships between partners that later buried the proverbial hatchet [11]. Other symbioses may be neither clearly antagonistic nor clearly mutualistic, but rather involve partners that either help or hurt one another depending on the environmental context or the genotypes of the partners involved [12–14]. Because symbiotic partners do not always have each other's best interests at heart, it may sometimes be necessary for symbionts to defend themselves from their partners, even in apparently mutualistic symbioses [15,16]. Conflict will often drive a need for symbionts to modify their own behaviour—or that of their partners—to coexist stably, and how symbiotic partners attune to one another is of special interest to understanding how symbioses start and which symbioses endure.

In this study, we focus on the social amoeba *Dictyostelium discoideum* and endosymbiotic bacteria in the genus *Paraburkholderia. Dictyostelium discoideum* is a normally unicellular eukaryote with a long history of being used as a model organism for scientists interested in its many multicellular behaviours. *Dictyostelium discoideum* and its relatives are facultatively multicellular organisms that spend most of their time as single, amoeboid cells, moving through forest soil, hunting bacteria, and reproducing vegetatively [17–19]. In adverse conditions, however, *D. discoideum* cells will aggregate into multicellular groups and undergo a sophisticated developmental process to form first a slug-like body that can travel to find a suitable site and then a fruiting body with which to remain dormant until they can disperse to greener pastures [20,21]. Formation of the fruiting body requires the sacrifice of some cells within the aggregate to form a stalk to hold the other cells aloft. These sacrificial stalk cells are akin to the somatic cells of more conventional multicellular organisms, performing some non-reproductive function (in this case, a structural one) so that other cells can reproduce. Many aspects of *D. discoideum's* development and evolution have been the focus of studies within a variety of fields [22,23].

Though they are the most obvious, the cells that die to form *D. discoideum's* stalk during the last stage of its development are not the only cells that perform somatic functions within *D. discoideum* aggregates. During the slug stage that precedes fruiting, another, smaller subpopulation of ‘sentinel cells' circulate within the aggregate collecting foreign bacteria and toxins, and are eventually sloughed off the slug and left behind prior to fruiting [24]. Though the adaptive significance of the sentinel cells' efforts to clear bacteria and toxins from the slug is not known, it seems likely they serve as a primitive immune system for *D. discoideum*.

*Dictyostelium discoideum* interacts with a wide variety of soil bacteria in nature [25–28]. Some are its prey, some are its pathogens, and some lie somewhere in between. Among this latter category are *Paraburkholderia agricolaris, P. hayleyella* and *P. bonniea,* which persistently infect *D. discoideum* cells as intracellular passengers [29]. Though in many respects *Paraburkholderia* acts as a pathogen, reducing the apparent fitness of its host, infection by *Paraburkholderia* imbues *D. discoideum* with the ability to carry prey bacteria with it throughout the social stages of its life cycle [30–32]. This bacterial carriage can enable *D. discoideum* to disperse to prey-impoverished environments not available to uninfected *D. discoideum.* Under the right conditions, therefore, *Paraburkholderia* may have a net benefit for its hosts and behave more like a mutualistic symbiont rather than a pathogen [13].

The interaction between *D. discoideum* and these *Paraburkholderia* species has both positive and negative consequences for both participants, and is a rising model system in the study of the evolution of interspecific interactions [13,30,33–35]. A heretofore largely unexplored direction, however, is how infection by *Paraburkholderia* may modulate *D. discoideum's* interactions with other bacteria in the soil environment.

In this study, we examine *Paraburkholderia's* effect on *D. discoideum* sentinel cells, and explore the consequences of these effects on *D. discoideum's* interactions with other bacterial pathogens that it might encounter in its natural habitat. This work builds on a previous study where we discovered that wild *D. discoideum* isolates infected by *Paraburkholderia* produce fewer sentinel cells than uninfected isolates [36]. If in fact sentinel cells perform an important immune function for *D. discoideum,* it seems intuitive that any disruption of sentinel cell function could render *D. discoideum* hosts more sensitive to toxins and pathogens. However, earlier work suggests that *Paraburkholderia* infection may actually increase hosts' resistance to ethidium bromide, despite interfering with the sentinel cells that would otherwise remove such toxins from *D. discoideum* aggregates. We hypothesized that it might provide a similar protective effect against intracellular pathogens that might otherwise threaten it and its hosts' fitness. In this study, we take advantage of recent advances in our understanding of the diversity of *Paraburkholderia* to further explore the consequences of its relationship with its *D. discoideum* hosts.

## 2. Material and methods

### 2.1. Culture conditions for *Dictyostelium discoideum* clones and bacteria symbionts

We used *D. discoideum* clones collected from Virginia, Texas and North Carolina. We grew *D. discoideum* clones from frozen spore stocks on SM/5 nutrient agar plates (2 g glucose, 2 g Oxoid bactopeptone, 2 g Oxoid yeast extract, 0.2 g MgSO_4_, 1.9 g KH_2_PO_4_, 1 g K_2_HPO_4_ and 15.5 g agar per litre double-distilled water (DDH_2_O)) and food bacteria *Klebsiella pneumoniae* at room temperature (22°C). We obtained *K. pneumoniae* from the Dicty Stock Center. *Klebsiella pneumoniae* was streaked onto SM/5 plates from frozen stocks and allowed to grow until stationary phase. We prepared *K. pneumoniae* bacterial suspensions with an optical density (OD) of A600 1.50 in KK2 buffer (2.25 g KH_2_PO_4_ and 0.67 g K_2_HPO_4_ per litre DDH_2_O) using a BioPhotometer (Eppendorf, New York). The *D. discoideum* clones and specific symbionts used in these experiments are included in table 1. We removed (cured) *Paraburkholderia* from the infected clones using either ampicillin-streptomycin or tetracycline antibiotic treatment. We verified *Paraburkholderia* removal using polymerase chain reaction (PCR) with *Paraburkholderia* specific primers [30].

**Table 1.** Table of strains.

clone (*D. discoideum*)	infection type	location collected	GPS coordinates	symbiont (*Paraburkholderia*)
QS70	infected	Houston Arboretum, TX	29°46′ N, 95°27′ W	*P. agricolaris* (PaQS70)
QS159	infected	Mt. Lake Biological Station, VA	37°21′ N, 80°31′ W	*P. agricolaris* (PaQS159)
QS161	infected	Mt. Lake Biological Station, VA	37°21′ N, 80° 31′ W	*P. agricolaris* (PaQS161)
NC21	infected	Linville Falls, NC	35°57′ N, 81°57′ W	*P. agricolaris*
QS70c	cured	Houston Arboretum, TX	29°46′ N, 95°27′ W	none
QS159c	cured	Mt. Lake Biological Station, VA	37°21′ N, 80°31′ W	none
QS161c	cured	Mt. Lake Biological Station, VA	37°21′ N, 80°31′ W	none
NC21c	cured	Linville Falls, NC	35°57′ N, 81°57′ W	none
QS11	infected	Mt. Lake Biological Station, VA	37°21′ N, 80°31′ W	*P. hayleyella* (PhQS11)
QS21	infected	Mt. Lake Biological Station, VA	37°21′ N, 80°31′ W	*P. hayleyella* (PhQS21)
QS22	infected	Mt. Lake Biological Station, VA	37°21′ N, 80°31′ W	*P. hayleyella* (PhQS22)
QS23	infected	Mt. Lake Biological Station, VA	37°21′ N, 80°31′	*P. hayleyella* (PhQS23)
QS11c	cured	Mt. Lake Biological Station, VA	37°21′ N, 80°31′	none
QS21c	cured	Mt. Lake Biological Station, VA	37°21′ N, 80°31′ W	none
QS22c	cured	Mt. Lake Biological Station, VA	37°21′ N, 80°31′ W	none
QS23c	cured	Mt. Lake Biological Station, VA	37°21′ N, 80°31′ W	none
QS17	naturally uninfected	Mt. Lake Biological Station, VA	37°21′ N, 80°31′ W	none
QS154	naturally uninfected	Mt. Lake Biological Station, VA	37°21′ N, 80°31′ W	none
QS157	naturally uninfected	Mt. Lake Biological Station, VA	37°21′ N, 80°31′ W	none
QS160	naturally uninfected	Mt. Lake Biological Station, VA	37°21′ N, 80°31′ W	none

### 2.2. Visualizing sentinel cells in slug trail assay

To determine if *D. discoideum* sentinel cell numbers are reduced by *Paraburkholderia* presence, we used four clones colonized with *P. agricolaris* and four clones colonized with *P. hayleyella*. These clones include QS70, QS159, QS161 and NC21 for *P. agricolaris* and QS11, QS21, QS22 and QS23 for *P. hayleyella*. We used the same eight clones cured of their *Paraburkholderia* infections as our uninfected control to compare against the infected *D. discoideum* clones.

We adapted methods from Brock *et al*. [36] to visualize and collect sentinel cells by staining *D. discoideum* isolates with a low dose of ethidium bromide (EtBr), an intercalating agent that interferes with nucleic acid synthesis and is commonly used as a fluorescent tag [37]. Using a low dose of ethidium bromide allowed us to visualize sentinel cells, which pick up the chemical and fluoresce, without increasing cell death [36]. However, sentinel cell identify is thus confounded with ethidium bromide treatment (though at a dose that should not have major effects). To prepare ethidium bromide-treated plates, we used 50 × 15 mm Petri plates with non-nutrient agar (9.9 g KH_2_PO_4_ monobasic, 1.78 g Na_2_HPO_4_ dibasic and 15.5 g agar per litre DDH_2_O) containing 1.0 µg per ml EtBr. Sixty millilitres of non-nutrient agar was poured first and allowed to set. We laid three microscope slides (3″ × 1″ × 1 mm) touching each other on top of the cooled agar. Then the slides were embedded in the agar by adding 25 ml of the same non-nutrient agar on top of the slides.

To set up the migration plates, we prepared a concentrated *K. pneumoniae* bacteria suspension. We used an overnight bacterial culture started from a single colony, and grown in Luria broth (10 g tryptone, 5 g Oxoid yeast extract and 10 g NaCl per litre DDH_2_O) shaking at 25°C. Next day, we pelleted the overnight culture by centrifugation at 10 000*g* for 5 min at 4°C discarding the supernatant. The bacterial pellet was resuspended and washed in KK2 buffer (2.25 g KH_2_PO_4_ and 0.67 g K_2_HPO_4_ per litre DDH_2_O). We resuspended the final pellet in a small volume of KK2. The bacterial suspension was diluted accordingly to obtain an OD A600 of 35.00 using a BioPhotometer (Eppendorf, New York).

We collected *D. discoideum* spores in KK2 buffer and determined the spore count using a haemacytometer and light microscope. We suspended 2.0 × 10^5^ spores in 200 µl of prepared bacterial suspension, and 50 µl of the mixture was dispensed in a line parallel to the embedded microscope slides on one edge of the plate. The spore mixture was allowed to dry. The plate was wrapped in aluminium foil with a small hole poked on the opposite side of the line where the spores were deposited. The plates were placed so the hole faced a source of light, towards which the slugs would migrate, and were stored at room temperature (22°C) for 168 h to allow for sufficient slug migration across the ethidium bromide starving agar Petri plate.

To visualize sentinel cells present in slug trails, we excised the embedded microscope slides and placed microscope slide coverslips (24 × 60 mm) on top of the slug trails on the agar. These trails were imaged using a Nikon A1Si laser scanning confocal microscope (Nikon, Tokyo), at 10× magnification under UV light (Texas red filter, *λ* = 561.3 nm). We used the ‘Scan Large Image' function in the software program (NIS Elements Advanced Research v. 4.12.01) to capture an image of the slug trails over a large span of area. This function captures multiple images over the selected area and stitches the images together (10% blend). We then used the ‘Annotations and Measurements' tool to measure the length of the sectioned trails from which sentinel cells were counted. Present in the slug trails were both single and clumped groups of sentinel cells. We counted sentinel cells in clumped groups as an estimate based on the size of one sentinel cell.

### 2.3. Colonizing uninfected hosts with *Paraburkholderia* and migration assay

To test if colonization with *Paraburkholderia* affects sentinel cell number, we first needed to determine what proportion of *Paraburkholderia* to mix with the food bacteria *K. pneumoniae*. Previously, we had determined that sufficiently high infectious doses of *Paraburkholderia* are toxic enough to prevent hosts from forming slugs or fruiting bodies (data not shown).

We prepared bacterial suspensions of *K. pneumoniae, P. agricolaris* BaQS159 and *P. hayleyella* BhQS11 at OD600 = 35.00 using the method described above. We then combined suspensions of food bacteria and either *P. agricolaris* or *P. hayleyella* at different ratios to compare the effects of different infectious doses. We performed 10-fold serial dilutions to test for slug formation from 1% *Paraburkholderia* + 99% *K. pneumoniae* down to 0.0001% *Burkholderia* + 99.9999% *K. pneumoniae.* The low percentage of *Paraburkholderia* was achieved by diluting the *Paraburkholderia* bacterial suspension down to a lower OD such that sufficient volume could be pipetted. We did not obtain adequate slug formation until we reduced the percentage of *Paraburkholderia* to 0.001% and 0.0001%. We followed the same steps to plate spores on ethidium bromide plates as described in the visualizing sentinel cells assay above. We used 100% *K. pneumoniae* as our control.

After the slugs were allowed to migrate and fruit, we tested sori to determine if bacteria carriage was induced successfully in naturally uninfected clones. We adapted methods of the spot test described in Brock *et al*. [34]. From the fruiting bodies formed at the ends of the slug trails, we randomly picked up individual sori using a filtered pipette tip. Each sorus was transferred onto SM/5 nutrient agar plates as individual spots. We incubated the plates at room temperature (22°C) for 2 days, examined for bacteria growth, and recorded the number of positive spots of bacterial growth.

### 2.4. Bead uptake assay

To determine if *Paraburkholderia*-infected sentinel cells are able to function as well as those from uninfected hosts, we counted the uptake of 0.5 µm diameter fluorescent latex beads in sentinel cells present in disassociated slugs. We used three types of host clones consisting of four naturally uninfected, three infected by *P. agricolaris*, and three infected by *P. hayleyella*. To remove extracellular bacteria, we washed log phase amoebae with KK2 buffer. These amoebae were then allowed to develop on filters until they formed aggregates. We collected aggregates with a pipette tip and disassociated cells by pipetting the collected aggregates several times. Disassociated cells were then mixed with fluorescent beads at a 1 : 10 ratio of cells to beads. Sentinel cells were identified by their ability to take up beads. We counted the number of beads present in each of 10 sentinel cells selected haphazardly for each *D. discoideum* clone in each set for a total of 100 sentinel cells.

### 2.5. Pathogen fitness assay

To assess fitness effects associated with carriage of *Paraburkholderia*, we chose two pathogenic bacteria species known to infect *D. discoideum* intracellularly (*Staphylococcus aureus* Rosenbach (Wichita) ATCC 29213, and *Salmonella enterica* ATCC 14028) [38,39]. For this assay, we tested four hosts naturally infected with *P. agricolaris*, four hosts cured of their *P. agricolaris*, four hosts naturally infected with *P. hayleyella*, four hosts cured of their *P. hayleyella*, and four naturally uninfected hosts. Table 1 for specific clone identities. Each clone was grown on either 100% *St. aureus* (Gram-positive pathogen), *Sa. enterica* (Gram-negative pathogen), or *K. pneumoniae* (good-food control). We used total spore production as our measure of host fitness. To set up each assay, we plated 2 × 10^5^ spores of each clone in each condition onto SM/5 agar plates in triplicate. All clones formed fruiting bodies within 2–3 days. We collected spores separately from two of the plates 5 days after fruiting using the method previously described in Brock *et al*. [34]. Briefly, we collected spores by washing plates with KK2 buffer supplemented with 0.01% NP-40 alternative (Calbiochem). Then, we counted spores using a haemacytometer and a light microscope.

### 2.6. Statistical analyses

We performed statistical analyses in R (v. 3.6.3). To compare the means of different groups, we fit models followed by pairwise contrasts calculated with the *emmeans* package [40] using fdr to adjust for multiple comparisons. Sentinel cell data was collected from slug trails with multiple measures from each trail and multiple trails for each clone. To account for this nested structure of sentinel cell counts, we included the random effects of trail nested within clone in linear mixed models (LMM) using the nlme package [41]. We also log transformed sentinel cell counts to reduce the skew from high counts. For our bead results, we fit generalized linear mixed models (GLMM) in the lme4 package [42] with a Poisson link function and clone as a random effect.

To measure how pathogens affected spore production, we used a generalized least-squares (GLS) model. Because infections with different pathogens resulted in groups with different variances, we weighted observations using the varIdent function in the nlme package. To reanalyse the spore data in Brock *et al*. [36] that tested the effect of a high dose of ethidium bromide, we used a LMM. To account for the two technical replicates used in this experiment, we included clone as a random effect. We scaled spore production values by subtracting the mean and dividing by the standard deviation.

For both our pathogen and ethidium bromide models, we estimated effects relative to the control condition (grown with *K. pneumoniae* food bacteria or without ethidium bromide), where hosts are not expected to suffer reduced spore production. This effect relative to the control will also capture the inedibility of pathogens, which is a consequence of pathogens being able to evade phagocytosis [43]. We are thus measuring the effect of some stressor (infection/inedibility or toxin) relative to healthy hosts. We are mostly interested in the interaction effects between *Paraburkholderia* infection status (infected or cured) and stressors (pathogen infection or toxins). These effects would indicate that *Paraburkholderia* infection (or curing) protects or causes increased harm for hosts when exposed to a stressor.

## 3. Results

### 3.1. Are symbiont *Paraburkholderia* the causal agents for immune-like sentinel cell number reduction?

**Figure 1.**
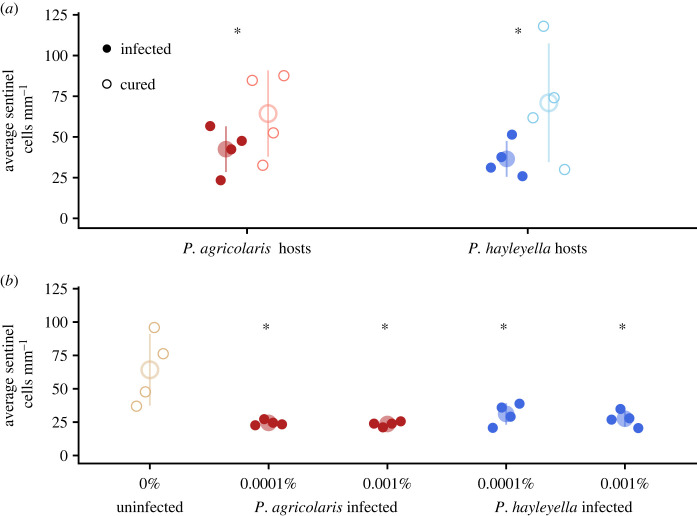
*Paraburkholderia* infection lowers the number of sentinel cells in *D. discoideum* hosts. (*a*) Infected *D. discoideum* clones have significantly fewer sentinel cells than the same clones cured of *Paraburkholderia* by antibiotics. The small points represent the average number of sentinel cells counted per mm in individual trails for each clone. (*b*) Naturally uninfected *D. discoideum* that are infected with *Paraburkholderia* have fewer sentinel cells even when infected at extremely low doses. Large points and lines show the mean and standard deviation. Asterisks show significant differences (*p* ≤ 0.05). Statistical comparisons in (*b*) are relative to uninfected controls.

Both curing hosts of their *Paraburkholderia* and infecting naive hosts showed that *Paraburkholderia* are responsible for changes to sentinel cells. Curing hosts of *P. agricolaris* infections increased the number of sentinel cells by 35% (ratio = 0.649, s.e. = 0.072, d.f. = 113, *p* = 0.002; figure 1*a*). Curing hosts of *P. hayleyella* infections increased the number of sentinel cells by 40% (ratio = 0.600, s.e. = 0.0664, d.f. = 113, *p* < 0.001). Infecting naturally uninfected (naive) hosts reduced the number of sentinel cells by 50% or more for both 0.001% and 0.0001% infection doses (figure 1*b*). Infecting hosts with *P. agricolaris* at 0.0001% (ratio = 0.389, s.e. = 0.042, d.f. = 129, *p* < 0.001) and 0.001% (ratio = 0.398, s.e. = 0.043, d.f. = 129, *p* < 0.001) resulted in about 60% fewer sentinel cells. Infecting hosts with *P. hayleyella* at 0.0001% (ratio = 0.504, s.e. = 0.055, d.f. = 129, *p* < 0.001) and 0.001% (ratio = 0.468, s.e. = 0.051, d.f. = 129, *p* < 0.001) resulted in about 50% fewer sentinel cells. Infecting hosts with different doses did not affect sentinel cell number for hosts infected with *P. agricolaris* (ratio = 0.979, s.e. = 0.105, d.f. = 129, *p* = 0.845) or hosts infected with *P. hayleyella* (ratio = 1.077, s.e. = 0.117, d.f. = 129, *p* = 0.550).

### 3.2. Is the function of sentinel cells from infected hosts impaired?

**Figure 2.**
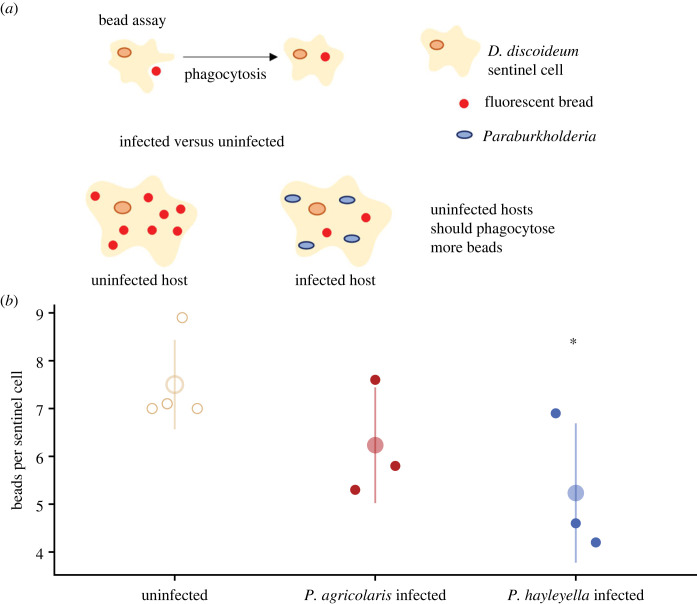
Sentinel cells from infected slugs are less functional than sentinel cells from uninfected slugs. (*a*) Schematic of fluorescent bead assay. (*b*) Number of beads phagocytosed by uninfected sentinel cells, sentinel cells infected with *P. agricolaris*, and sentinel cells infected with *P. hayleyella*. Small points represent the average number of beads for each clone (counted from 10 individual sentinel cells). Large points and lines show the mean and standard deviation. Asterisks show significant differences (*p* ≤ 0.05) relative to uninfected controls.

Because hosts infected with *Paraburkholderia* have fewer sentinel cells (figure 1), we suspected *Paraburkholderia* infection may also impact sentinel cell function. To test sentinel cell function, we measured the number of beads that sentinel cells were able to phagocytose. We found that sentinel cells from *P. agricolaris*-infected hosts take up fewer beads (about 17% less) compared with uninfected hosts (figure 2), but this difference was not significant (ratio = 0.830, s.e. = 0.098, *p* = 0.115) Sentinel cells from *P. hayleyella*-infected hosts take up about 30% fewer beads than uninfected hosts (ratio = 0.696, s.e. = 0.085, *p* = 0.006; figure 2). Thus, hosts infected by *P. hayleyella* have reduced sentinel cell function.

### 3.3. How does *Paraburkholderia* infection affect host response to toxicity and pathogens?

A prior study found that *Paraburkholderia* infection protected hosts when they were exposed to a toxically high (more than 10 times the dose used in this study) amount of ethidium bromide [36]. However, hosts in their natural soil environment are unlikely to come in contact with ethidium bromide. We instead sought a more natural stressor to understand how reduced sentinel cell function affected hosts. We suspected that reduced function of sentinel cells due to *Paraburkholderia* infection may make infected hosts more susceptible to harm from other pathogens.

**Figure 3.**
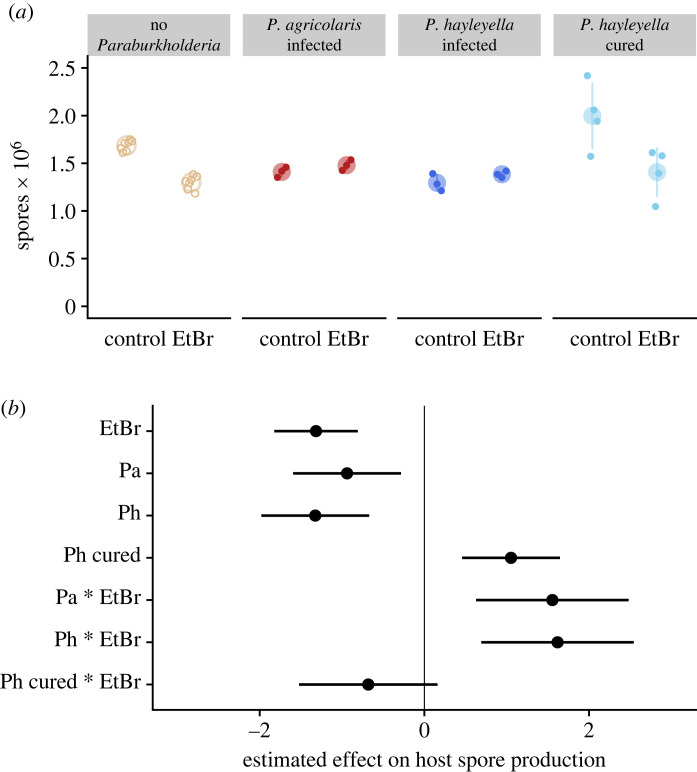
*Paraburkholderia* infection protects hosts from high-dose ethidium bromide. (*a*) Spore production from hosts with different infection statuses. These data are from Brock *et al*. [36] broken up by the species of infecting *Paraburkholderia*. Control *D. discoideum* were grown on starving agar without ethidium bromide. (*b*) Estimated effects of high-dose ethidium bromide, *Paraburkholderia* infection status, and interactions on host spore production. Host spore production in the model was scaled by subtracting the mean and dividing by the standard deviation.

We first reanalysed the data from Brock *et al*. [36] where infected, uninfected and cured hosts were grown on starving agar plates with or without toxic ethidium bromide (figure 3*a*). While the original study included hosts infected by both *P. agricolaris* and *P. hayleyella,* it did not draw a distinction between the two species. (Additionally, the dataset only included cured controls for strains infected by *P. hayleyella*). We estimated the effects on host spore production of ethidium bromide, infection, curing, and the interactions between these categories relative to uninfected controls that were not exposed to ethidium bromide. We describe the estimated effects from top to bottom in figure 3*b*. We found that ethidium bromide (EtBr) lowered host spore production (figure 3*a,b*; estimate = −1.316, 95% CI = [−1.824, −0.809], d.f. = 59). The harm of ethidium bromide was a similar magnitude as the characteristic cost of infection for both *P. agricolaris* (Pa, estimate = −0.939, 95% CI = [−1.594, −0.284], d.f. = 59) and *P. hayleyella* (Ph, estimate = −1.326, 95% CI = [−1.980, −0.671], d.f. = 59) [30]. Interestingly, curing hosts of their *P. hayleyella* increased spore production relative to uninfected hosts (Ph cured, estimate = 1.053, 95% CI = [0.458, 1.647], d.f. = 59). Validating the results of Brock *et al*. [36] that found a protective effect of *Paraburkholderia* infection, we found that hosts infected with *P. agricolaris* (Pa*EtBr, estimate =1.5547826, 95% CI = [0.629, 2.481], d.f. = 59) or *P. hayleyella* (Ph*EtBr, estimate =1.618, 95% CI = [0.692, 2.544], d.f. = 59) produced more spores when exposed to ethidium bromide than expected from the separate effects of *Paraburkholderia* infection and ethidium bromide. This effect is due to *Paraburkholderia* infection, at least for *P. hayleyella*, as these hosts cured of their symbionts did not deviate from the additive effects of curing and ethidium bromide (Ph cured*EtBr, estimate = −0.681, 95% CI = [−1.523, 0.160], d.f. = 59).

**Figure 4.**
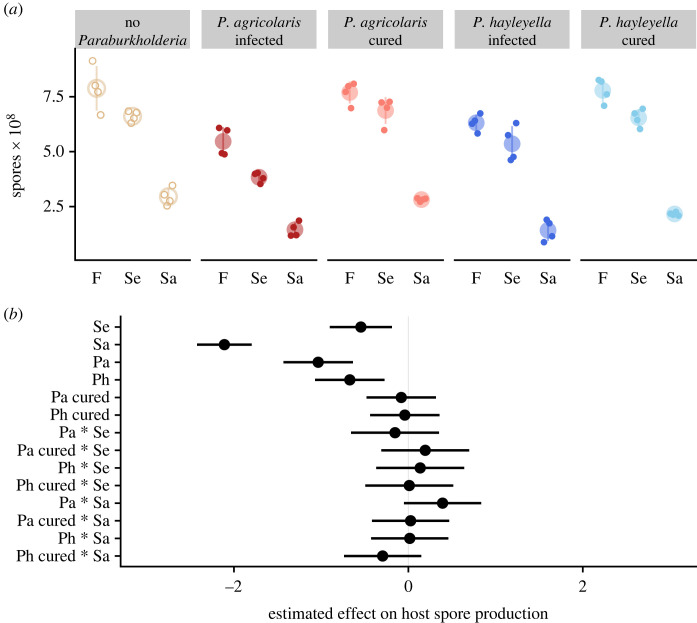
*Paraburkholderia* infection does not protect hosts from pathogenic *Salmonella enterica* (Se) or *Staphylococcus aureus* (Sa) bacteria. (*a*) Spore production of hosts when grown on food bacteria (F) or pathogenic *Salmonella enterica* (Se) or pathogenic *Staphylococcus aureus* (Sa). (*b*) Estimated effects (points) and 95% confidence intervals of *Paraburkholderia* infection (Pa, *P. agricolaris*; Ph, *P. hayleyella*), pathogen infection (Sa or Se) and co-infection (shown joined by *) on *D. discoideum* spore production. Spore production values were scaled by subtracting the mean and dividing by the standard deviation.

To address the possibility that *Paraburkholderia* infection makes hosts more prone to harm from a more natural stressor, we tested spore production in the presence of two pathogenic bacteria, *Salmonella enterica* and *Staphylococcus aureus*. We used *Klebsiella pneumoniae* as our control food bacteria for comparison. To measure the harm of infection with these other pathogens, we counted the spores produced by *D. discoideum* (figure 4*a*). We asked two questions: (i) Does infection with pathogens lower *D. discoideum* spore production relative to growing on food bacteria? (ii) Does infection with *Paraburkholderia* affect harm to hosts when infected with other pathogens?

To answer these questions, we fit a generalized least-squares model with interaction terms between each pathogen infection status (either *Sa. enterica* or *St. aureus*) and each *Paraburkholderia* infection status (either *P. agricolaris*, cured of *P. agricolaris*, *P. hayleyella* or cured of *P. hayleyella*) to *D. discoideum* spore production measures (figure 4*a*). We used this model to estimate the effects of pathogen infection, *Paraburkholderia* status, and co-infections (interactions) relative to uninfected *D. discoideum* that were grown on only food bacteria (figure 4*b*). Working down from the top of figure 4*b*, we found that *Sa. enterica* is moderately pathogenic (Se, estimate = −0.544, 95% CI = [−0.901, −0.188], d.f. = 60) while *St. aureus* is highly pathogenic, reducing host spore production almost fourfold (Sa, estimate = −2.110, 95% CI = [−2.423, −1.797], d.f. = 60) relative to *Sa. enterica*. *Paraburkholderia* infection by *P. agricolaris* (Pa, estimate = −1.034, 95% CI = [−1.432, −0.636], d.f. = 60) or *P. hayleyella* (Ph, estimate = −0.672, 95% CI = [−1.070, −0.273], d.f. = 60) is similarly pathogenic as *Sa. enterica*, and curing obviated this cost (figure 4*b*; cured Pa and cured Ph effects are essentially 0).

Co-infection by *Paraburkholderia* and a pathogen did not increase or decrease harm from pathogens beyond the additive effects of both kinds of infection (interaction terms with *s overlap 0). These results show that *Paraburkholderia* and pathogen infections both reduce *D. discoideum* spore production, but that *Paraburkholderia* does not make hosts more susceptible to harm by pathogens. On the other hand, *Paraburkholderia* also offers no protection against the pathogens we tested in contrast to the results with ethidium bromide.

## 4. Discussion

In this study, we investigated the effects of *Paraburkholderia* infection on the sentinel cells of *D. discoideum* hosts. Due to sentinel cells' presumed function as a primitive immune system within *D. discoideum* aggregates, we also explored the consequences of *Paraburkholderia* infection on *D. discoideum*'s sensitivity to toxins and pathogens.

*Dictyostelium discoideum* aggregates produce a subpopulation of sentinel cells which seem to serve as an innate immune system for the multicellular stages of its life cycle [24]. Like the cellular immune systems of more familiar multicellular organisms, sentinel cells circulate within *D. discoideum* aggregates, sequestering and disposing of potentially hazardous foreign material like toxins or pathogens. In a previous study, we observed that some wild *D. discoideum* isolates carrying certain *Paraburkholderia* bacteria through their social cycles also produced significantly fewer sentinel cells [36].

To explore the role of *D. discoideum's* intracellular symbiont *Paraburkholderia* on its host's sentinel cells, we compared the number of sentinel cells produced in slugs made by wild *D. discoideum* strains known to harbour *P. agricolaris* or *P. hayleyella* symbionts with slugs made by the same strains after antibiotic treatment. We found that antibiotically treated *D. discoideum*, cured of their normal symbionts, produced significantly more sentinel cells than infected cells (figure 1). When we then reinfected these cured strains with *P. agricolaris* or *P. hayleyella* in the laboratory, the effect was reversed and fewer sentinel cells were produced. These results establish a causal link between infection by *Paraburkholderia* and the previously observed reduced sentinel cell numbers in infected *D. discoideum* strains. *Paraburkholderia* infections reduced *D. discoideum* sentinel cell production even at very small infectious doses.

In addition to quantifying the reduction in sentinel cell numbers in infected *D. discoideum*, we also tested sentinel cell functionality by measuring sentinel cells' ability to sequester fluorescently labelled beads. We found that individual sentinel cells produced by infected *D. discoideum* sequestered significantly fewer beads than sentinel cells from uninfected *D. discoideum* (figure 2). We suspect that *D. discoideum* sentinel cells take up fewer beads because *Paraburkholderia* infection reduces their function. However, we cannot completely rule out that external *Paraburkholderia* are affecting bead engulfment. During the disassociation step, where *D. discoideum* cells in aggregates are separated, *Paraburkholderia* could be released. If these bacteria bind to beads and affect phagocytosis this could also explain our results. However, we think this explanation is less likely than infection lowering sentinel cell function. External bacteria should be rare in our experiments because infection doses were low and experiments were performed on filters without nutrients for bacterial proliferation.

Our results suggest that *D. discoideum* infected by *Paraburkholderia* not only produces fewer sentinel cells, but those that it does produce are less functional. Insofar as sentinel cells serve an important role in clearing potentially damaging foreign substances from multicellular *D. discoideum* aggregates prior to the production of fruiting bodies, we expected infected *D. discoideum* to have impaired immune function and be more sensitive to toxins or pathogens. To test this, we first reanalysed data measuring the toxic effect of high-dose ethidium bromide on spore production in infected and uninfected *D. discoideum* [36]. Uninfected *D. discoideum* exposed to toxic amounts of ethidium bromide produces fewer spores during its fruiting stage (figure 3), presumably reflecting death or reduced functionality of cells within the aggregate. By contrast, however, *D. discoideum* infected by *Paraburkholderia* produced similar numbers of spores with and without the presence of ethidium bromide. While infected *D. discoideum* produce fewer spores overall due to the effects of *Paraburkholderia* infection itself [30], they no longer appear to be sensitive to ethidium bromide's toxic effects. One potential explanation for this reduced toxicity is that *Paraburkholderia* themselves are taking up ethidium bromide and reducing exposure to their hosts.

Sentinel cells may represent an adaptation against threats posed by pathogens—either through infection or via the production of toxins. *Dictyostelium discoideum* is known to be susceptible to a wide variety of pathogens [44], which, combined with its similarities to human macrophages, has made it a common model for the study of pathogenesis and immunity [45]. Previous work suggests that sentinel cells probably serve a protective function against pathogens, presumably by sequestering and removing them from *D. discoideum* slugs as they do with ethidium bromide [24]. Sentinel cells were shown to upregulate their expression of the *tirA* and *tirB* genes, apparent homologues of genes known to be involved in animal and plant innate immunity signalling. Mutant strains lacking functional *TirA* showed increased sensitivity to the virulent pathogen *Legionella pneumophila*.

In the light of sentinel cells' potential immune function, we explored whether *Paraburkholderia's* effects on sentinel cell number and functionality increased *D. discoideum's* sensitivity to other pathogens, as would be expected if sentinel cell activity played a key role in immunity. We exposed uninfected amoebae and amoebae carrying *Paraburkholderia* symbionts to *Staphylococcus aureus* and *Salmonella enterica*, two pathogens known to infect *D. discoideum* [38,39]*.* Unsurprisingly, *D. discoideum* grown on either pathogen produced fewer spores relative to more edible bacteria, reflecting *St. aureus* and *Sa. enterica's* negative effects. Despite its effects on sentinel cell number and functionality, however, infection by *Paraburkholderia* did not have a significant effect on *D. discoideum's* sensitivity to either tested pathogen (figure 4).

Our results suggest that *D. discoideum* infected by *Paraburkholderia* experiences reduced sentinel cell production and function. Building upon previous work, this study demonstrates that *Paraburkholderia* itself is responsible for changes in sentinel cells (rather than some strains of *D. discoideum* evolving reduced sentinel cells to facilitate symbiosis or for some other purpose). Naive *D. discoideum* strains with no known association with *Paraburkholderia* produce fewer sentinel cells when infected, and infected *D. discoideum* sentinel cell production is restored when *Paraburkholderia* symbionts are cleared by antibiotic treatment.

It is unclear whether *Paraburkholderia*'s effect on its host's sentinel cells is adaptive. Given that some evidence suggests sentinel cells may be involved in clearing bacteria from *D. discoideum* slugs, it is intuitive to speculate that *Paraburkholderia* could benefit from inhibiting them. Alternatively, *Paraburkholderia*'s effects on sentinel cells may be pleiotropic consequences of other traits. *Paraburkholderia*'s intracellular lifestyle is probably only possible because it can waylay the mechanisms by which *D. discoideum* phagocytoses and destroys its prey. A recent study [46] has shown that *Paraburkholderia* inhibit *D. discoideum* phagosomes from becoming acidic, potentially preventing digestion. If phagocytosis is also key to sentinel cell function during the slug stage, whatever mechanism *Paraburkholderia* uses to survive phagocytosis during its host's vegetative stage may inhibit sentinel cell function as a side effect.

Despite its effect on sentinel cell number and function, *Paraburkholderia* infection also has the apparently paradoxical effect of *reducing* its host's sensitivity to the toxin ethidium bromide. One possible explanation is that *Paraburkholderia* may neutralize ethidium bromide in a way which compensates for the presumed loss of *D. discoideum*'s own defences resulting from fewer, less functional sentinel cells. Many bacteria have metabolic capabilities unavailable to eukaryotes, and these form the basis for various other symbioses [47–49]. Previous work has suggested that *Paraburkholderia* and *D. discoideum* have a complex relationship with both antagonistic and cooperative elements, and it is likely that whether *Paraburkholderia* is a beneficial partner or a parasite depends on the specific context. If *Paraburkholderia* can protect its host from toxins, then the likelihood of encountering these toxins could play a role in whether *Paraburkholderia* behaves as a cooperative symbiont or a parasite.

By contrast, we did not observe any effect of *Paraburkholderia* infection on *D. discoideum's* sensitivity to the pathogens *St. aureus* or *Sa. enterica.* A previous study demonstrated that *D. discoideum* mutants lacking a functional *tirA* gene—normally highly expressed in sentinel cells—were extremely sensitive to virulent *L. pneumophila* [24]*.* This has been taken to imply an important role for sentinel cells in protecting *D. discoideum* from pathogens, but our results cast some doubt on this interpretation. *Paraburkholderia-*driven reductions in sentinel cell number and functionality did not seem to render *D. discoideum* any less capable of surviving *St. aureus* or *Sa. enterica* as would be expected if they served a vital immune function against these pathogens. It may be that sentinel cells' potential immune function is context specific, or that *D. discoideum* combats different pathogens in different ways. Alternatively, perhaps infected *D. discoideum* with fewer and less functional sentinel cells *are* more sensitive to pathogens but are compensated for somehow by the presence of *Paraburkholderia.* This compensation could result from competition between *Paraburkholderia* and pathogens that reduces pathogen density. Similar benefits from bacterial interactions inside hosts have been suggested for numerous other systems [50,51].

The specific role of *D. discoideum's* sentinel cells in defending against specific pathogens is not yet fully clear, but we consider it likely that they function as a simple immune system for the multicellular portions of *D. discoideum's* life cycle. Previous studies demonstrate that sentinel cells sequester and discard toxic ethidium bromide, which may suggest a more general role in combatting toxins produced by other microbes in *D. discoideum's* environment. If sentinel cells do have an immune function, then *Paraburkholderia's* ability to reduce sentinel cell production and functionality may be an adaptation to actively interfere with its host's defences.

Regardless of whether *Paraburkholderia* acts as a pathogen or a mutualist, this sort of immune interference has precedence in other systems. Pathogens, perpetually locked in antagonistic coevolutionary arms races with their hosts, have evolved myriad ways of interfering with every part of their hosts' immune systems [52,53]. While this sort of antagonism might be expected between foes, it is also known to occur in apparently cooperative interactions. Research on other host/symbiont systems ranging from plants and their root symbionts [54] to animals and their symbionts [55–59] suggests that modulation of the host's immune system—whether imposed by the host itself or by interference from the symbiont—is often critical for establishment and persistence of symbioses.

*Paraburkholderia*'s effect on *D. discoideum*'s sentinel cells adds an intriguing element to our understanding of their already complex relationship. This study has identified an additional cost of being infected by *Paraburkholderia*: reduced sentinel cell function. Further exploration into the mechanisms by which *Paraburkholderia* infects and persists within *D. discoideum* will help further develop the system as a model for symbiosis, cooperation and antagonism, and provide insights into how immune systems respond to symbionts.

## Data Availability

All data and code for this project are included as electronic supplemental material [60] and publicly available at https://gitlab.com/treyjscott/sentinel_cell_project.
